# Odontogenic Factors Associated with Maxillary Sinus Schneiderian Membrane Thickness and their Relationship to Chronic Sinonasal Symptoms: An Ambispective Cohort Study

**DOI:** 10.3390/diagnostics13162710

**Published:** 2023-08-20

**Authors:** Maha Alghofaily, Noura Alsufyani, Riyadh I. Althumairy, Amal AlSuhaibani, Fatimah Alfawzan, Lama AlSadhan

**Affiliations:** 1 Restorative Dental Sciences, College of Dentistry, King Saud University, Riyadh 11461, Saudi Arabia; 2 Oral & Maxillofacial Radiology, Oral Medicine and Diagnostic Sciences Department, College of Dentistry, King Saud University, Riyadh 11461, Saudi Arabia; 3 School of Dentistry, Department of Medicine and Dentistry, University of Alberta, Edmonton, AB T6G 2R3, Canada; 4 College of Dentistry, King Saud University, Riyadh 11461, Saudi Arabia; amal.suh@gmail.com (A.A.); alfawzan.fatimah@gmail.com (F.A.); lamalsadhan@gmail.com (L.A.)

**Keywords:** odontogenic sinusitis, Schneiderian membrane, sinonasal symptoms, cone-beam computed tomography

## Abstract

Odontogenic sinusitis is a common maxillary sinus disease. It develops due to the violation of the Schneiderian membrane due to pathological, iatrogenic, or traumatic causes from dental and dentoalveolar structures. The aim of this cohort study was to investigate local and systemic factors associated with Schneiderian mucosal thickening (MT) in patients referred for evaluation of apical periodontitis (AP) and examine their relationship with chronic sinonasal symptoms. Cone-beam computed tomography (CBCT) scans of 197 patients referred for evaluation of endodontic diseases were reviewed. Mucosal thickening in relation to the affected tooth was measured in the coronal section in millimeters at the maximum area perpendicular to the bone. Based on this measurement, the sinus floor was categorized for MT as present (>1 mm) or absent (<1 mm). The sociodemographic and clinical characteristics of the study participants were assessed and compared according to the presence or absence of MT. Furthermore, the relationship between odontogenic sinusitis and chronic sinonasal symptoms was assessed using a chronic sinusitis survey. Male patients had a higher MT than female patients. The presence of periapical lesions and inadequate endodontic treatment were significantly associated with MT. The multivariate logistic regression model showed that the presence of AP increased the odds of MT by approximately 2.5 times (*p* = 0.028), and nasal obstruction was significantly higher in patients diagnosed with mucosal thickness at different times of their follow-up period (*p* = 0.018). MT was 2.5 times more likely in the presence of apical periodontitis, and nasal obstruction was the most significant factor associated with the presence of MT.

## 1. Introduction

Maxillary sinus pathology is one of the most prevalent illnesses and significantly impacts health [1]. The disease may be caused by different etiological factors, including rhinogenic, odontogenic, traumatic, allergic, and neoplastic factors [2]. Chronic maxillary sinusitis (MS) is episodes of sinus membrane inflammation that covers the paranasal cavity, persisting for at least 8–12 weeks, with signs and symptoms such as nasal obstruction, nasal discharge, facial pain or pressure, and/or loss of smell [3]. The appearance of a normal sinus membrane may not be observed by radiographs; however, when infection or allergy occurs, leading to the thickening of the membrane, it becomes visible on images [4].

A significant proportion of MS is associated with odontogenic infection from posterior maxillary teeth as a result of close anatomic proximity to the sinuses [5,6]. Many clinical studies have documented varying rates of odontogenic maxillary sinusitis, ranging from 10% to 86% [7,8,9]. One of the significant factors associated with the risk of maxillary sinusitis is apical periodontitis (AP) [10]. The progression of periapical lesions (PA) from the posterior maxillary teeth can lead to inflammatory changes in the mucosal lining of the maxillary sinus, subsequently leading to sinusitis [5]. Most of the cases with changes in the maxillary sinus floor were of odontogenic origin [11]. These changes are related to the anatomical relationship between the maxillary posterior teeth and the maxillary sinus, leading to structural changes in the Schneiderian mucosal sinus membrane and possibly the entire sinus. These changes present radiographically as partial or complete sinus opacification [12]. Such thickening indicates pathological alteration and carries the risk of developing MS at the clinical level [13]. Generally, the maxillary sinus is considered normal when a uniform thickening of the membrane measuring about 1 mm [12,14] or 2 mm is observed [15,16].

Understanding the relationship between sinus disease and odontogenic infections is necessary for proper diagnosis and management. The use of cone-beam computed tomography (CBCT) for clinical diagnosis in different dental disciplines is clinically valuable for the detection of disease. This is particularly important in revealing sinus diseases affected by AP [17]. Although many other pathological conditions can be associated with the thickening of the mucosal membrane, which is difficult to distinguish radiographically, many otolaryngologists and rhinologists recognize that odontogenic sinusitis should always be considered first in the presence of chronic sinusitis; however, such evaluations are rarely described in routine clinical practice [18,19]. This may result in persistent chronic sinusitis even after local treatment, resulting in significant long-term symptoms and the need for medication [20,21]. Main symptoms including facial pain or pressure, nasal congestions, rhinorrhea, and smell disorder were related to odontogenic sinusitis [22]. The majority of patients with odontogenic sinusitis complained of rhinorrhea and smell disorder [23]. However, these symptoms do not always distinguish sinusitis of odontogenic origin from other sinusitis [22]. The imaging modality, especially the CBCT, is considered the gold standard for the determination of AP and the thickening of the membrane. It offers high-resolution images in multiple planes and allows for the detailed examination of the maxillary sinus and the detection of any sinonasal inflammation [24].

Despite the tremendous evidence linking odontogenic infections and dental or surgical procedures with sinusitis, still physicians overlook it [13]. Most cases frequently remain unrecognized by radiologists, dentists, and ear, nose, and throat (E.N.T) specialists [25]. Therefore, more information describing the potential clinical factors associated with Schneiderian mucosal thickening (MT) is warranted. In addition, no reports have looked at the status of chronic sinusitis in the presence or absence of MT.

This study aimed first to identify local and systemic factors associated with MT in patients referred for endodontic evaluation and second to examine the relationship between odontogenic sinusitis and chronic sinonasal symptoms.

## 2. Materials and Methods

### 2.1. Study Design and Population

The present study, designed as an observational cross-sectional cohort study, included two phases:

- Review of dental records and CBCT scans of patients who presented with endodontic evaluation at the University Hospital between January 2017 and December 2020.
- Follow-up survey of the chronic sinusitis status using the chronic sinusitis survey (CSS).

Ethical approval was obtained from the Institutional Review Board (project no. E-21-5974). STROBE guidelines were followed to ensure the proper reporting of this observational study [26].

Inclusion and exclusion criteria:
Inclusion criteria were as follows:

1. Patients must have endodontic disease including symptomatic apical periodontitis, asymptomatic apical periodontitis, acute apical periodontitis, or chronic apical periodontitis in the maxillary posterior teeth.
2. The radiographic appearance of the teeth must be presented in high-quality technical images with appropriate sharpness, density, and contrast to visibly show the maxillary posterior tooth apices and sinus floor.

Exclusion criteria were as follows:

1. Completely edentulous maxilla.
2. Presence of motion or beam-hardening artifacts in the maxillary periapical areas.
3. Teeth without complete data in the records.

Data collection:

For all posterior maxillary teeth included in the study, the following clinical elements from patients and dental records were collected:

1. Patient age and sex
2. Tooth type and number (molar, premolar)
3. Extent (single or multiple teeth)
4. Periodontal status (healthy periodontium with < 4 mm pocket depth or periodontal disease with > or =4 mm periodontal pockets)
5. Endodontic status, including the presence/absence of periapical lesions (PA) and adequacy of endodontic treatment
6. Self-reported medical history

Radiographic assessment:

The maxillary sinuses were evaluated by an oral and maxillofacial radiologist for sinus variables on CBCT scans. The scans were obtained using a Planmeca ProMax 3D Plus (Planmeca Co., Ltd., Helsinki, Finland). The field of view ranged from small to large according to the original area of interest and the parameters used were 90 kVp, 11 mA, 12.146 s, and 200 μm voxel size. Each scan was reviewed in all sections, including coronal, axial, and sagittal views, to assess individual areas. The MT was measured in coronal sections by measuring the mucosal thickness of the sinus floor in relation to the involved teeth in millimeters at the maximum area perpendicular to the bone. 

**Figure 1:**
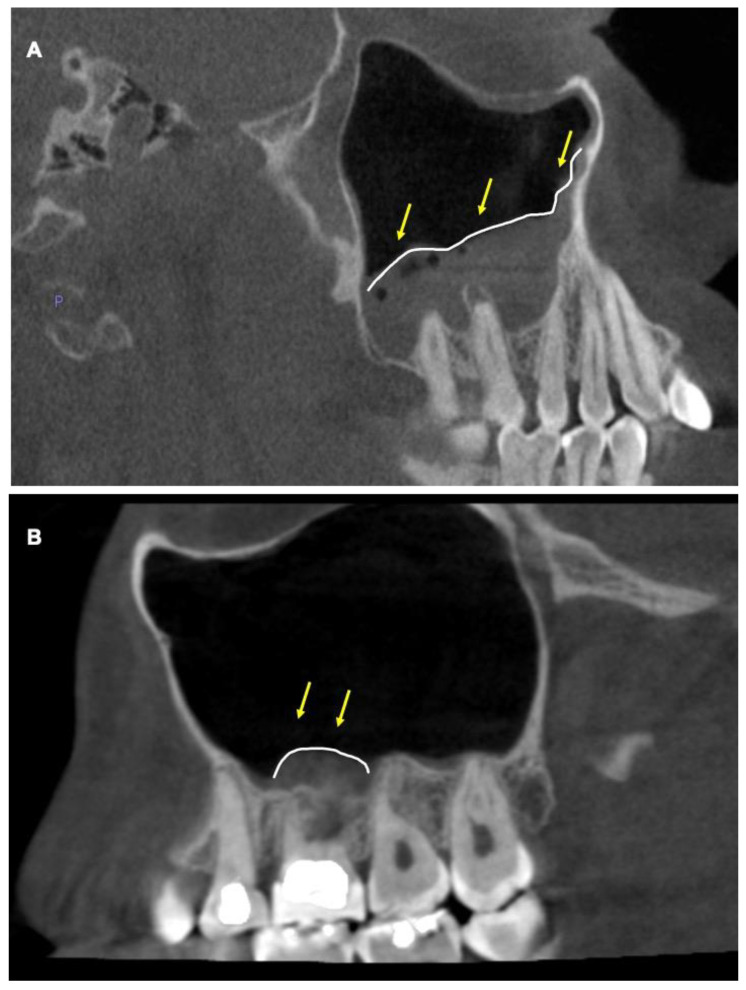
**Cone-beam CT images showing mucosal thickening in maxillary sinus.** (**A**) Sagittal view showing generalized mucosal thickening of the left maxillary sinus that extends beyond the maxillary molar area. Yellow arrows pointing to the area. (**B**) Sagittal view of localized mucosal thickening. Yellow arrows showing mucosal thickening of the left maxillary sinus localized to the maxillary molar area.

Based on this measurement, the sinus floor was categorized for MT as present (>1 mm) or absent (<1 mm). Furthermore, the presence of MT was classified as localized or generalized as described previously [12] (Figure 1A,B). 

Apical lesions and endodontic treatments were evaluated by endodontists using the Planmeca Romexis^®^ software 4.6.0 R (Planmeca, Finland) on periapical digital radiographs. The following information was recorded:

1. Presence/absence of periapical lesions based on the periapical index [27]: PAI score categorized as absence (representing a PAI score of 1 or 2) or presence (representing a PAI score of 3, 4, or 5).
2. The quality of endodontic treatment was categorized as adequate (representing adequate density of root canal filling and length of 0–2 mm from the radiographic apex with adequate coronal coverage) or inadequate (presence of voids, missed canal or short filling, and no coronal coverage).

### 2.2. Follow-Up Survey

This survey was used to assess the clinical symptoms of persistent chronic sinusitis. Patients who met the inclusion criteria were contacted by phone and asked about their status of chronic sinusitis using the chronic sinusitis survey (CSS). This survey is a valid, disease-specific questionnaire to assess the health status of chronic rhinosinusitis [28]. The questionnaire was translated into Arabic and validated, and open access was provided to researchers [29]. The survey consisted of two parts as follows: symptom- and medication-based sections. The symptoms assessed were sinus headache or facial pain, nasal drainage, and nasal obstruction. The medication-based section included the use of antibiotics, nasal spray, and sinus medications. This section asks questions based on the duration of symptoms over 8 weeks. Patients were asked about the time of symptoms, if they evolved during the time of their endodontic evaluation, and if they were asymptomatic for long periods.

#### Statistical Analysis

The variables analyzed were mucosal thickness, periapical lesions of the teeth and roots, tooth type, patient sex and age, systemic factors, and CSS scores. Data were descriptively analyzed (frequencies, means, and standard deviations) to summarize the sociodemographic and clinical characteristics of the study subjects. The associated variables among age, sex, systemic factors, periapical lesions, periodontal health, quality of endodontic treatment, and tooth type in relation to the absence or presence of MT were assessed using the chi-square test. Mean CSS scores were compared between sub-groups (presence or absence of MT) using a one-way ANOVA. Logistic regression was used to calculate the odds ratio (OR) and assess the relative risk concerning local and systemic factors. All variables were dichotomized for binary and logistic regression analyses. All analyses were performed using IBM SPSS Statistics software (version 28) (SPSS, Armonk, NY, USA). The level of significance was set at *p* < 0.05 and was considered statistically significant.

## 3. Results

Of the 595 CBCT scans, 197 were included; 112 (56.9%) were male and 85 (43.1%) were female, with a mean age of 43 ± 11.6 years. Around 60.9% of the patients were more than 40 years old while the rest were less than 40 years. Table 1 shows the distribution of the sociodemographic and clinical characteristics of the study participants and their association with the presence of MT. MT was present in 162 (82%) of the samples, with a mean of (6.1 ± 5.8) mm. Most patients presented with generalized MT rather than localized MT (57.4% vs. 24.9%, respectively). The second molar was the tooth most associated with the presence of MT compared to the other teeth. PA lesions were detected in 132 (67%) samples. The quality of endodontic treatment was adequate in 113 (57.4%) samples. The presence of PA lesions and inadequate endodontic treatment was significantly associated with MT. Men had a significantly higher incidence of MT compared to women.

We found that 89.4% of patients with PA lesions had MT, while (67.7%) of the patients without PA lesions had MT, and the difference in the proportion was statistically significant (*p* < 0.001; Table 1). The presence of MT was also found to be significantly associated with the quality of endodontic treatment. We found that 91.4% of the patients with inadequate quality of endodontic treatment had MT, while 76.1% of the patients with adequate quality of endodontic treatment had MT (*p* = 0.011). Men were significantly higher in proportion (86.6%) compared to women (76.5%) in terms of having MT (*p* = 0.049; Table 1). 

Multivariate analysis of the logistic regression model performed with all variables entered (type tooth, periodontal status, presence of PA lesion, quality of endodontic treatment, age, sex, smoking, diabetes mellitus, CVD) revealed that only the presence of PA lesions increased the odds of MT by approximately 2.5 times (*p* = 0.028). No other variables were associated with MT (Table 2).

Only 107 respondents (54%) responded to the CSS survey by phone; the majority (85%) of them reported their symptoms at the time of their presentation with endodontic diseases. Sixty-three patients (58.9%) had seasonal headaches and experienced pain for 7−8 weeks. The number of patients who reported nasal drainage and breathing difficulties was 68 and 56 patients, respectively. A total of 38 patients with MT used antibiotics, nasal spray, and sinus medication over 8 weeks (Table 3).

**Figure 2:**
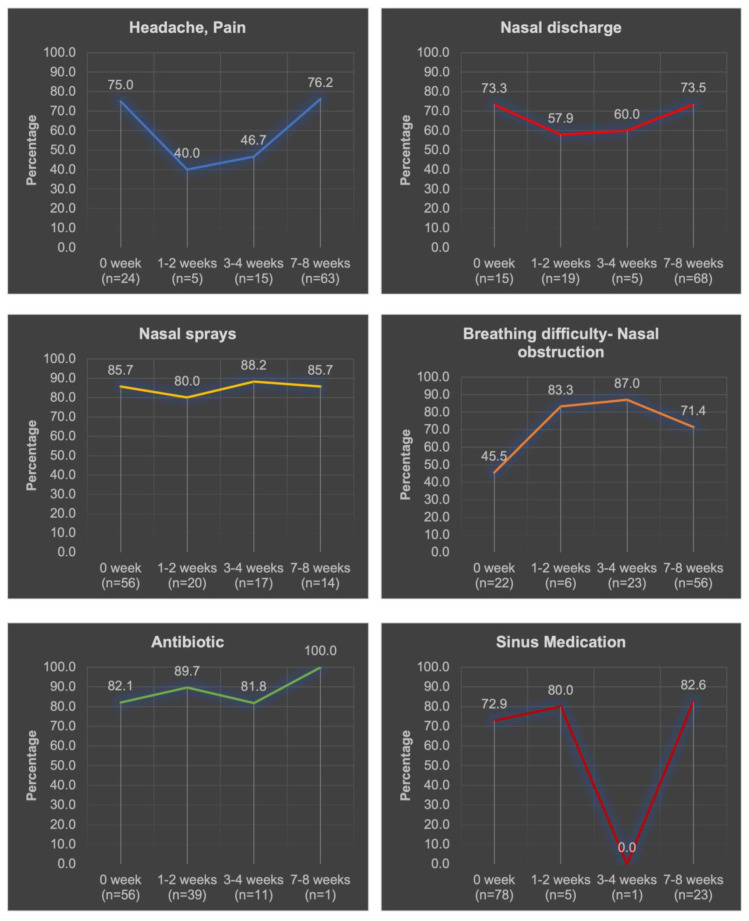
Distribution of study participants’ responses with “Yes “symptoms to the chronic sinusitis survey (*n* = 107). Total number of patients who experienced different symptoms over the 8 weeks is demonstrated below each duration between (*n*=).

Figure 2 showed the different symptoms patients experienced over the 8 weeks in relation to MT. A significant increase in breathing difficulty/nasal congestion over the time duration was observed in relation to MT (*p* = 0.018). 

The total scores of the patients for the CSS questions were not significantly different between those with or without MT. The mean total score was 17.08 (±5.9) in patients with MT and 15.59 (±6.1) in patients without MT (*p* > 0.05).

## 4. Discussion

The use of CBCT imaging technology in endodontics has helped evaluate and diagnose pathological conditions in the posterior maxilla and interpret the relationship between dental pathology and the involved sinus [30,31]. Persistent chronic apical periodontitis can cause the thickening of the Schneiderian membrane and maxillary sinusitis [32,33]. This radiographic study evaluated odontogenic sinusitis and its associated clinical factors using CBCT scans. Our findings indicated a high prevalence of sinus MT in endodontic patients, representing 82.2% of the samples selected. The most prevalent condition reported in this study was generalized MT (57%), followed by localized MT (24%). We used the methodology proposed by Nascimento [12] who considered the presence of MT when a thickness of >1 mm was present. Our findings are consistent with those of other studies in which sinus disease was reported in 85% of maxillary sinuses related to odontogenic origin, and most of the cases were classified as generalized (62.6%) vs. localized (24%) [12]. Previous CBCT studies have reported a prevalence of sinus MT between 38−82% using various thresholds of 1−3 mm of thickening [7,16,34]. This variation was attributed to the population and age of the participants, as well as various diagnostic techniques [35]. However, there is still no consensus on the extent to which MT is considered pathological [12,31]. Various studies measured the mean thickness of the Schneiderian membrane using fresh cadavers that showed no sign of maxillary sinusitis and reported about 0.30 to 0.80 mm [36] or 0.02–0.35 mm [37]. Similarly, Aimetti et al. found a mean thickness value of 0.97–0.36 mm in samples analyzed from healthy participants [38]. Because of this controversy, we have reported both thicknesses in the present study. Our study observed a high prevalence of sinus disease, which can be attributed to the sample selected, composed of CBCT images of patients referred from the endodontic department to evaluate apical periodontitis.

In this study, there was a significant difference in the presence of MT in patients with apical lesions compared to those without lesions. As a result of the very close relationship between the root apices of maxillary posterior teeth and the sinus floor [39], various dental conditions, such as apical pathology, extraction, placement of a dental implant, and trauma, are well known to violate the integrity of the Schneiderian membrane and increase the risk of maxillary sinusitis [40]. This persistent, underlying, long-standing microorganism of odontogenic origin can spread to the maxillary sinus and manifest itself as symptoms and thickening of the Schneiderian membrane. Endodontic infections mainly rely on primary (bacterial invasion and colonization from necrotic pulp tissue) or secondary (persistent microorganisms in the root canal system of endodontically treated teeth) infections [41]. The prolonged action of the causative factors of microgames can act directly and indirectly to affect the sinus structure and induce inflammation without perforating the sinus cortical bone [39]. In other cases in which the molars are in close proximity to the sinus and the periapical bone is very thin, perforation of the maxillary sinus may also occur [42]. Bauer described the extension of periapical inflammation to the maxillary sinus in 1943 [43] using a cadaver model under microscopic evaluation. The inflammatory mediators induced by infection can spread via the bone marrow, lymph nodes, and blood vessels of the maxillary sinus [43]. Chronic periapical lesions in both primary [15,16,33] and secondary [39] endodontics may lead to new periosteal bone formation and changes in the thickness of the Schneiderian membrane [33]. Bivariate analysis also showed that the quality of endodontic treatment had a significant effect on the presence of MT. A previous study also indicated that the quality of root canal treatment shown by good length and good quality filling is associated with the absence of sinus abnormalities [44]. In addition, the quality of root canal filling is associated with apical pathology [45]. The percentage of cases reported in our study with inadequate root canal treatment was approximately 30% and was associated with the presence of PA lesions. This is in agreement with previous studies that showed the importance of complete cleaning and disinfection followed by an adequate apical seal and coronal restoration to control microorganisms [45,46,47]. It has been shown that the quality of endodontic treatment is significantly correlated with the presence and absence of apical pathology [45]

Demographic factors were not significantly associated with MT, except for sex. In this study, males presented more frequently with MT. However, multivariate regression analysis did not show a significant effect. Previous studies have shown that odontogenic-related sinusitis is more common in males than in females [48,49]. Demographic factors vary among the different reports in the literature [7,16,50]. Shabahang et al. reported that the prevalence of MT was associated with older age groups (>40 years) than with younger age groups [15].

The second molar was the tooth that was most associated with MT. This can be attributed to the proximity of the apices to the floor of the maxillary sinus. Estrela et al. reported the shortest distance from the apex to the maxillary sinus found in the maxillary second molar compared to the other maxillary teeth [51]. A similar study observed that the mesiobuccal root of the maxillary second molar was closely related to the maxillary sinus [52].

In the present study, different factors were found to play a role in the prevalence of MT. The odds of MT were higher with periodontal pockets > 4 mm, inadequate endodontic treatment, the presence of PA lesions, age < 40 years, male sex, smoking, and diabetes (regression table). Multivariate analysis showed that the only statistically significant factor associated with MT was the presence of PA lesions in CBCT. More than 80% of cases with MT were associated with PA lesions. The severity of MT increases with the prevalence of AP, as shown in previous studies [32,35,44,52] Consistent with these findings, a systematic review and meta-analysis reported a high OR of MT in the presence of PA lesions [31].

Chronic sinusitis is diagnosed by the presence of two of the following persistent symptoms lasting a minimum of 8–12 weeks: nasal obstruction, nasal drainage, facial pain, and olfactory dysfunction [10]. In conjunction with diagnostic aids, nasal endoscopy and imaging are used to confirm mucosal changes within the sinus and/or osteomeatal complex. In this study, we surveyed patients over the phone using the CSS symptoms scale to evaluate sinusitis symptoms. Only half of the patients responded to the survey. Patients with and without MT were compared using the CSS symptoms scale. In general, patients with MT had a significantly higher percentage of nasal congestion. There was no significant difference between the scores. Hoskison et al. reviewed the incidence of odontogenic sinusitis and its clinical features and reported that out of 26 patients, rhinorrhea and smell disorder were the most common presenting complaints in patients with odontogenic sinusitis, found in 81 and 73 per cent, respectively. In addition, pain and nasal obstructions were reported [23]. Our findings on the mean between the groups may be underestimated. However, our explanation is that patients with mucosal thickness might have had resolved symptoms as many of them underwent endodontic treatment after CBCT imaging. A recent study by Park et al. evaluated the radiographic changes in sinus mucosal thickness in patients with MT of odontogenic origin before extraction using CBCT. The treatment of the extraction of compromised teeth and drainage during lateral sinus augmentation showed a successful reduction in MT. In addition, sinonasal symptoms were improved significantly postoperatively [13].

This study has several limitations that should be acknowledged. We attempted to correlate the radiographic findings and clinical symptoms of patients with MT. Changes in the Schneiderian membrane may be transient during active disease. As this study was a retrospective study, it allowed us to link the association between endodontic diseases and sinus abnormalities. It was unknown whether all patients had undergone dental treatment after the diagnosis of the disease, and it was difficult to identify all patients included in our sample. Future clinical studies should be performed using CBCT data from long-term follow-up cases, and the maxillary sinus should be examined in otolaryngologic and clinical dental practices.

## 5. Conclusions

In conclusion, MT was prevalent and was 2.5 times more likely to present with AP. The presence of nasal obstruction was the most significant factor associated with the presence of MT. Proper diagnosis and management require the collaboration of dental and E.N.T specialists.

## Figures and Tables

**Table 1:** Association of sociodemographic and clinical characteristics of study subjects associated with MT (*n* = 197).

Characteristics	*n* (%)	Presence of MT		*p*-Value
		Yes, *n* (%)	No, *n* (%)	
**Age groups**				
<40	77 (39.1)	61 (79.2)	16 (20.8)	
>40	120 (60.9)	101 (84.2)	19 (15.8)	0.242
**Sex**				
Male	112 (55.9)	97 (86.6)	15 (13.4)	
Female	85 (43.1)	65 (76.5)	20 (23.5)	0.049 *
**Smoking status**				
Yes	23 (11.7)	19 (82.6)	4 (17.4)	
No	174 (88.3)	143 (82.2	31 (17.8)	0.541
**Diabetes mellitus**				
Yes	15 (7.6)	13 (86.7)	2 (13.3)	
No	182 (92.4)	149 (81.9)	33 (18.1)	0.407
**CVD**				
Yes	9 (4.6)	6 (66.7)	3 (33.3)	
No	188 (95.4)	156 (83)	32 (17)	0.222
**Extent**				
Single	159 (80.7)	129 (81.1)	30 (18.9)	
Multiple	38 (19.3)	33 (86.8)	5 (13.2)	0.285
**Periodontal status**				
PD < 4	162 (82.2)	133 (82.2)	29 (17.9)	
PD ≥ 4	35 (17.8)	29 (82.9)	6 (17.1)	0.568
**Presence of PA lesion**				
Yes	132 (67)	118 (89.4)	14 (10.6)	
No	65 (33)	44 (67.7)	21.(32.3)	<0.001*
**Quality of endodontic treatment**				
Adequate	113 (57.4)	86 (76.1)	27 (23.9)	
Inadequate	58 (29.4)	53 (91.4)	5 (8.6)	0.011 *
No treatment	26 (13.2)			

* Asterisks denote statistically significant differences (*p* < 0.05). *n*: overall sample; CVD: cardiovascular diseases; PD: periodontal pocket.

**Table 2:** Regression analysis to determine factors associated with the presence of mucosal thickening.

Variable	OR (95% CI)	*p*-Value
**Age groups**	0.569 (0.238–1.362)	0.205
**Sex**	2.254 (0.954–5.328)	0.064
**Smoking status**	1.139 (0.323–4.012)	0.840
**Diabetes mellitus**	1.234 (0.238–6.400)	0.802
**CVD**	0.303 (0.045–2.061)	0.222
**Extent** (Single/Multiple)	2.254 (0.684–7.428)	0.182
**Periodontal status**	1.258 (0.408–3.880)	0.689
**Presence of PA lesion**	2.762 (1.115–6.845)	0.028 *
**Quality of endodontic treatment**	2.472 (0.800–7.635)	0.116

* Asterisks denote statistically significant differences (*p* < 0.05). OR: odds ratio; CI: confidence interval.

**Table 3:** Distribution of study subjects’ responses to chronic sinusitis survey (*n* = 107).

	MT	Duration of Symptoms over 8 Weeks								*p*-Value
		0 Week		1–2 Weeks		3–4 Weeks		7–8 Weeks		
		*n*	%	*n*	%	*n*	%	*n*	%	
**Headache, pain**	No	6	25.0	3	60.0	8	53.3	15	23.8	0.058
	Yes	18	75.0	2	40.0	7	46.7	48	76.2	
	Total	**24**	**22.4**	**5**	**4.7**	**15**	**14.0**	**63**	**58.9**	
**Nasal drainage**	No	4	26.7	8	42.1	2	40.0	18	26.5	0.562
	Yes	11	73.3	11	57.9	3	60.0	50	73.5	
	Total	**15**	**14.0**	**19**	**17.8**	**5**	**4.7**	**68**	**34.5**	
**Breathing difficulty, nasal obstruction**	No	12	54.5	1	16.7	3	13.0	16	28.6	0.018 *
	Yes	10	45.5	5	83.3	20	87.0	40	71.4	
	Total	**22**	**20.6**	**6**	**5.6**	**23**	**21.5**	**56**	**52.3**	
**Antibiotic**	No	10	17.9	4	10.3	2	18.2	-	-	0.726
	Yes	46	82.1	35	89.7	9	81.8	1	100.0	
	Total	**56**	**52.3**	**39**	**36.4**	**11**	**10.3**	**1**	**0.9**	
**Nasal sprays**	No	8	14.3	4	20.0	2	11.8	2	14.3	0.905
	Yes	48	85.7	16	80.0	15	88.2	12	85.7	
	Total	**56**	**52.3**	**20**	**18.7**	**17**	**15.9**	**14**	**13.1**	
**Sinus medication**	No	10	12.8	1	20.0	1	100.0	4	17.4	0.103
	Yes	68	87.2	4	80.0	-	-	19	82.6	
	Total	**78**	**72.9**	**5**	**4.7**	**1**	**0.9**	**23**	**21.5**	

* Asterisks denote statistically significant differences (*p* < 0.05).

## Data Availability

The data presented in this study are available upon request from the corresponding author.
